# 超高效液相色谱-四极杆/静电场轨道阱质谱快速测定保健食品中13种*β*-受体阻滞剂

**DOI:** 10.3724/SP.J.1123.2023.06006

**Published:** 2023-11-08

**Authors:** Tonghui LI, Feng FENG, Yiming SHEN, Feng ZHANG

**Affiliations:** 1.中国检验检疫科学研究院食品安全研究所, 北京 100176; 1. Institute of Food Safety, Chinese Academy of Inspection and Quarantine, Beijing 100176, China; 2.河南省食品和盐业检验技术研究院, 国家市场监管重点实验室(食品安全快速检测与智慧监管技术), 河南 郑州 450000; 2. Henan Institute of Food and Salt Industry Inspection Technology, Key Laboratory of Food Safety Quick Tesking and Smart Superbision Technology for State Market Regulation, Zhengzhou 450000, China

**Keywords:** 超高效液相色谱-四极杆/静电场轨道阱质谱法, *β*-受体阻滞剂, 保健食品, ultra performance liquid chromatography-quadrupole/electrostatic field orbitrap mass spectrometry, *β*-blockers, health foods

## Abstract

建立了基于超高效液相色谱-四极杆/静电场轨道阱质谱快速测定保健食品中13种*β*-受体阻滞剂的分析方法,并对其质谱裂解规律进行了分析研究。实验对质谱条件、色谱条件、提取溶剂及基质效应等进行了详细探究,使用甲醇对样品进行稀释提取,高速离心、超声处理,用Acquity UPLC BEH C18柱(100 mm×2.1 mm, 1.7 μm)分离,以乙腈和0.1%(v/v)甲酸水溶液作为流动相进行梯度洗脱,采用正离子模式检测,数据采集使用一级母离子全扫描和数据依赖的二级离子扫描(Full MS/dd-MS^2^)模式,在10 min内实现了保健食品中13种*β*-受体阻滞剂的分离和高精度一级、二级扫描,得到准确的质量数和准确碎片离子信息。通过方法学验证,13种*β*-受体阻滞剂在0.5~100 μg/L内线性关系良好,相关系数(*r*)均≥0.9912,检出限为1~10 μg/kg。空白加标样品中13种*β*-受体阻滞剂的平均回收率为75.3%~108.4%,相对标准偏差为0.9%~10.0%(*n*=6)。用本方法对市售的30批次保健食品进行筛查,均未检出13种*β*-受体阻滞剂。该方法检测速度快,准确性强,灵敏度高,可用于保健食品中*β*-受体阻滞剂的快速测定。

## Abstract (en)

A method based on ultra performance liquid chromatography-quadrupole/electrostatic field orbitrap mass spectrometry was developed for the rapid determination of 13 *β*-blockers in health foods. The MS fragmentation pathways of the analytes were subsequently investigated. The optimal MS conditions, extraction solvents, mobile phases, and matrix effects were evaluated in detail. The samples were extracted with methanol, filtered by high-speed centrifugation and ultrasonic treatment, and then separated on an Acquity UPLC BEH C18 column (100 mm×2.1 mm, 1.7 μm) with gradient elution using acetonitrile and 0.1% (v/v) formic acid aqueous solution as mobile phases. MS analysis was conducted in positive-ion mode, and the data were collected using full mass and data-dependent MS^2^ scans (Full MS/dd-MS^2^). The efficient separation and high-precision primary and secondary scanning of the 13 *β*-blockers in health foods were realized within 10 min, and accurate mass numbers and fragment-ion information were obtained. The methodological validation showed good linear relationships in the range of 0.5-100 μg/L, with correlation coefficients (*r*)≥0.9912. The limits of detection ranged from 1 to 10 μg/kg. When the standard substances were added to the blank sample in the amount of 10-200 μg/kg, the recoveries were in the range of 75.3%-108.4%, and the relative standard deviations ranged from 0.9% to 10.0% (*n*=6). The method was used to screen 30 batches of commercially available health foods, and none of the 13 *β*-blockers was detected. The proposed method is fast, accurate, and sensitive, and can be used for the rapid determination of *β*-blockers in health foods.

当前,随着健康意识的增强,人们更倾向通过饮食来达到降血压的目的,许多声称具有降血压功能的保健食品也不断涌向市场^[1⇓-3]^。然而,为了追求利益,部分不法生产者会在一些保健食品中非法添加*β*-受体阻滞剂类药物以吸引消费者而获利。不当食用含有*β*-受体阻滞剂类药物的食品将会导致心肌耗氧量和血管阻力增加,氧自由基和心肌细胞凋亡^[4,5]^,对人体带来严重的健康风险。因此,开发一种保健食品中*β*-受体阻滞剂类药物的检测方法,并对市售的相关产品展开风险监测,以确保市场流通保健食品的质量安全成为当务之急。

当前,对于*β*-受体阻滞剂的检测,报道的方法主要有气相色谱-质谱法(GC-MS)^[6⇓-8]^、毛细管电泳法^[9,10]^、荧光光谱法^[11]^、酶联免疫法^[12]^、液相色谱-串联质谱法(LC-MS/MS)^[13⇓⇓⇓⇓-18]^、高效液相色谱法(HPLC)^[19,20]^、解吸附电晕束离子源耦合离子阱质谱法(DCBI-MS)^[21]^等,这些方法覆盖的*β*-受体阻滞剂范围较少,且在应用于食品样品检测时都面临着基质干扰无法有效消除的难题。近年来,高分辨质谱作为一种食品中非法添加物筛查的有效手段,已在食品检测中广泛使用,但目前还少有报道使用高分辨质谱进行保健食品中*β*-受体阻滞剂检测的方法,难以满足实际检测的需要。

本研究通过优化色谱及质谱条件,建立了一种超高效液相色谱-四极杆/静电轨道阱高分辨质谱快速测定食品中13种*β*-受体阻滞剂的分析方法,并成功应用于实际样品的测定,方法快速便捷,准确率高,为监管部门对保健食品中非法添加的分析检测提供了重要支撑。

## 1 实验部分

### 1.1 仪器、试剂与材料

Q Exactive四极杆/静电场轨道阱质谱系统及Dionex UltiMate 3000超高效液相色谱系统(美国Thermo Fisher公司);分析天平XP 105 (瑞士Mettler公司); Milli-Q Advantage A10超纯水机(美国Millipore公司)。

13种*β*-受体阻滞剂标准物质:阿替洛尔(atenolol, CAS号29122-68-7)、醋丁洛尔(acebutolol, CAS号37517-30-9)、普萘洛尔(propranolol, CAS号318-98-9)、纳多洛尔(nadolol, CAS号42200-33-9)、拉贝洛尔(labetalol, CAS号36894-69-6)、比索洛尔(bisoprolol, CAS号66722-44-9)、塞利洛尔(celiprolol, CAS号56980-93-9)、卡维地洛(celiprolol, CAS号72956-09-3)、阿罗洛尔(arotinolol, CAS号68377-92-4)、艾司洛尔(esmolol, CAS号81147-92-4)、美托洛尔(metoprolol, CAS号37350-58-6)、奈必洛尔(nebivolol, CAS号99200-09-6)、贝凡洛尔(bevantolol, CAS号59170-23-9),纯度均大于99%,均购自天津阿尔塔科技有限公司;甲醇、乙腈(LC-MS级,德国Merck公司);甲酸(LC-MS级,赛默飞世尔科技有限公司)。

30批食品样品:2022年7月分别从电商及实体店铺购买,其中10批次为固态片剂食品,20批次为液态口服液类样品。

### 1.2 实验条件

#### 1.2.1 溶液配制

分别准确称取/移取13种*β*-受体阻滞剂标准物质适量,用甲醇溶解并定容至10 mL,配制成质量浓度为100 μg/mL的标准储备液,-18 ℃避光保存。

分别准确吸取13种标准储备液适量,用甲醇定容至10 mL,配制成质量浓度为1.0 μg/mL的混合标准溶液,-18 ℃避光保存。

标准工作液:根据需要吸取适量混合标准溶液,用甲醇稀释成所需浓度,现配现用。

#### 1.2.2 样品制备

对于固态片剂样品,取适量混匀研磨后,精密称取1 g(精确至0.001 g),置于50 mL具塞离心管中,加入甲醇10 mL,涡旋1 min使其混合均匀,在25 ℃下通过超声波(40 kHz, 250 W)提取30 min,放冷至室温。提取后,混合物以8000 r/min离心5 min,收集上清液至20 mL容量瓶中,用上述方法重复提取一次,合并2次提取液,定容至刻度,摇匀。过0.22 μm有机滤膜后上机测定。

对于液态样品,经充分混匀后准确吸取1.0 mL置于50 mL具塞离心管中,加甲醇40 mL,在25 ℃下超声(40 kHz, 250 W)提取15 min,放冷至室温。用甲醇定容,过0.22 μm有机滤膜后上机测定。

#### 1.2.3 色谱条件

色谱柱:Waters Acquity UPLC BEH C18(100 mm×2.1 mm, 1.7 μm);流动相:0.1%(v/v)甲酸水溶液(A)-乙腈(B);流速0.2 mL/min,进样体积3 μL,柱温35 ℃。梯度洗脱程序:0~3.0 min, 98%B; 3.0~10.0 min, 98%B~5%B; 10.0~15.0 min, 5%B; 15.0~20.0 min, 5%B~98%B。

#### 1.2.4 质谱条件

离子源:加热电喷雾离子源(HESI),正离子模式;喷雾电压:3.0 kV;毛细管温度:320 ℃;加热器温度:50 ℃;鞘气:氮气,流速为40 arb;辅助气:氮气,流速为5 arb;扫描模式:一级母离子全扫描和数据依赖的二级子离子扫描(Full MS/dd-MS^2^)模式。一级母离子全扫描分辨率:7×10^4^半峰宽(FWHM);质谱扫描范围:*m/z* 100~1200;自动增益控制(AGG)及最大注入时间(IT)分别为1×10^6^、100 ms。数据依赖二级子离子扫描分辨率:1.75×10^4^ FWHM;触发阈值:1×10^5^;归一化碰撞能量设定为15%、30%、45%。13种*β*-受体阻滞剂的质谱分析参数见表1。

**表 1:** 13种*β*-受体阻滞剂的质谱参数

Compound	Molecular formula	t_R_/ min	Structural formula	Theoretical [M+H]^+^	Measured [M+H]^+^	Fragment ions (m/z)
Atenolol	C_14_H_22_N_2_O_3_	6.23	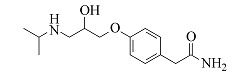	267.1703	267.1694	225.1227, 208.0975, 116.1077, 98.0973
Nadolol	C_17_H_27_NO_4_	6.77	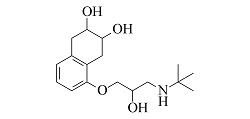	310.2013	310.2017	205.0868, 149.0240, 119.7833
Acebutolol	C_18_H_28_N_2_O_4_	7.25	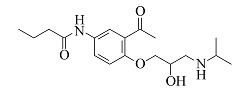	337.2122	337.2108	319.2004, 260.1272, 116.1070, 98.0967
Arotinolol	C_15_H_21_N_3_O_2_S_3_	7.26	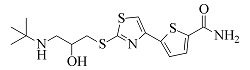	372.0869	372.0856	316.0232, 298.9968, 74.0607
Metoprolol	C_15_H_25_NO_3_	7.39	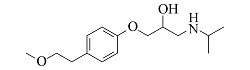	268.1907	268.1898	191.1060, 159.0799, 116.1069, 98.0967
Celiprolol	C_20_H_33_N_3_O_4_	7.55	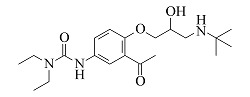	380.2544	380.2527	307.1648, 251.1021, 119.7495
Esmolol	C_16_H_25_NO_4_	7.57	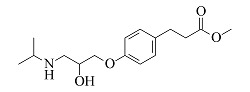	296.1856	296.1846	219.1008, 145.0644, 116.1070, 98.0967
Labetalol	C_19_H_24_N_2_O_3_	7.77	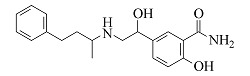	329.1860	329.1848	311.1743, 294.1479, 162.0544, 91.0546
Bisoprolol	C_18_H_31_NO_4_	7.86	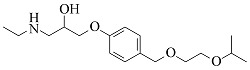	326.2326	326.2314	116.1070, 98.0967, 74.0607
Propranolol	C_16_H_21_NO_2_	8.01	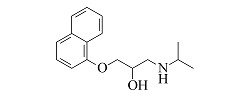	260.1645	260.1637	183.0799, 157.0644, 116.1070, 98.0967
Bevantolol	C_20_H_27_NO_4_	8.13	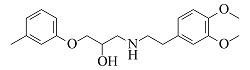	346.2013	346.2000	165.0906, 150.0671
Carvedilol	C_24_H_26_N_2_O_4_	8.35	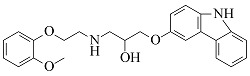	407.1965	407.1953	283.1430, 224.1273, 100.0759
Nebivolol	C_22_H_25_F_2_NO_4_	8.55	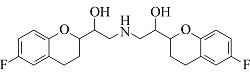	406.1824	406.1814	388.1701, 151.0550, 123.0603

## 2 结果与讨论

### 2.1 质谱条件的优化

本研究采用Q Exactive高分辨质谱的Full MS/dd-MS^2^模式进行13种*β*-受体阻滞剂的分析,这些化合物的一级离子及二级碎片离子信息见表1。通过使用目标化合物的精确分子离子质量数,结合二级碎片离子谱的分析,能够实现在没有标准品的情况下,对样品中有无上述13种*β*-受体阻滞剂类化合物进行快速筛查。使用超高效液相色谱-四极杆/静电场轨道阱质谱对13种*β*-受体阻滞剂的分子离子提取色谱图见图1。

**图1:**
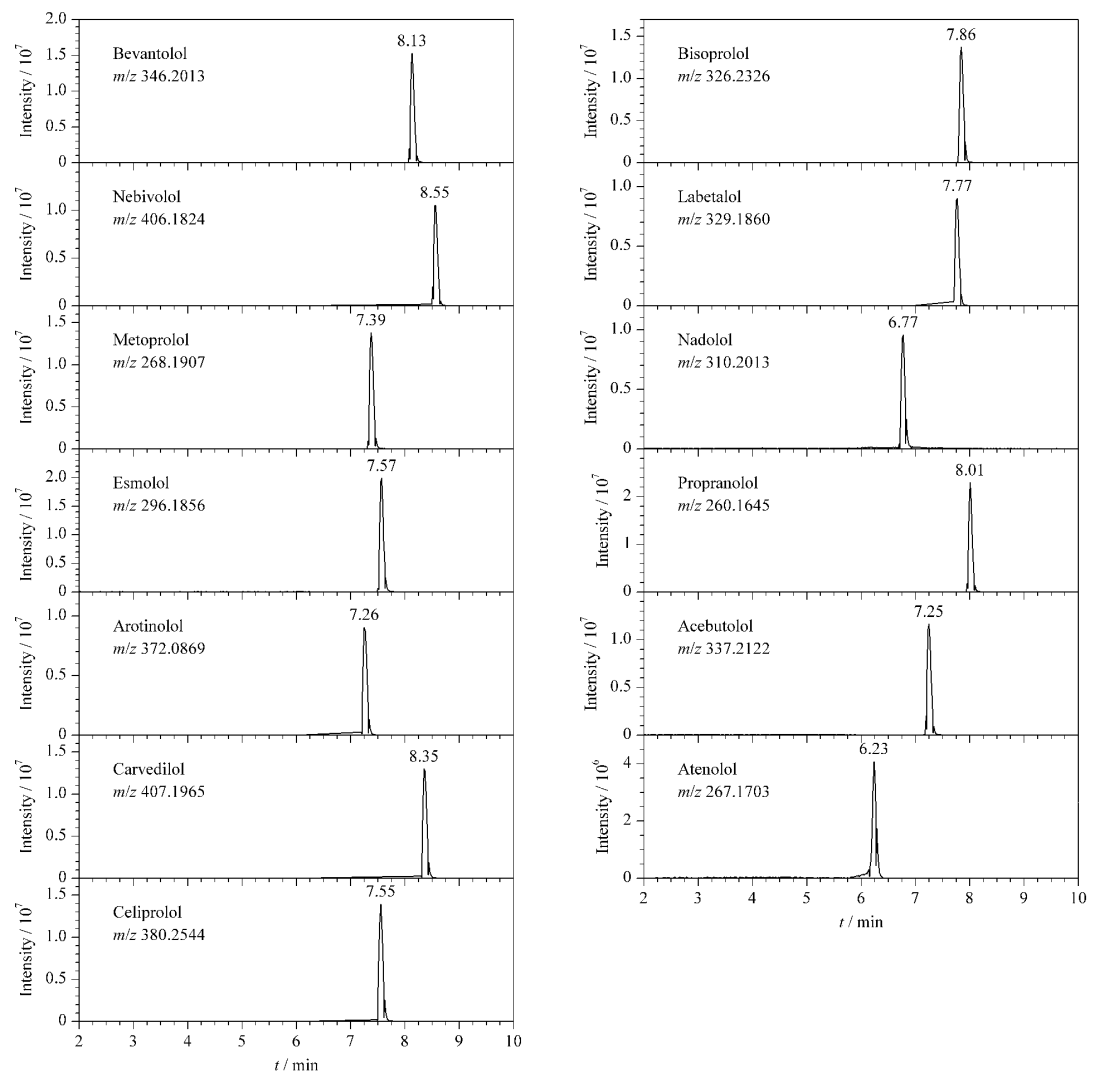
13种*β*-受体阻滞剂的提取离子色谱图

### 2.2 流动相及提取溶剂的选择

实验考察了0.1%(v/v)甲酸水溶液-甲醇、0.1%(v/v)甲酸水溶液-乙腈、0.02 mol/L乙酸铵溶液-乙腈、0.02 mol/L乙酸铵溶液(含0.1%(v/v)甲酸)-乙腈等流动相体系在最优的分离条件下各化合物的峰形及分离情况,发现0.1%(v/v)甲酸水溶液-乙腈作为流动相,梯度洗脱后13种化合物在10 min内能够得到较好的分离。

参考已有相关文献^[22⇓-24]^报道,实验考察了以甲醇、乙腈、80%(v/v)甲醇水溶液、1%(v/v)乙酸甲醇、1%(v/v)乙酸乙腈为提取溶剂时空白加标样品中13种*β*-受体阻滞剂类的加标回收率,如图2所示。结果表明,甲醇作为提取溶剂时样品中13种*β*-受体阻滞剂的回收率在80.4%~116.4%范围内,提取效果整体较优,故本实验选择甲醇为提取溶剂。

**图2:**
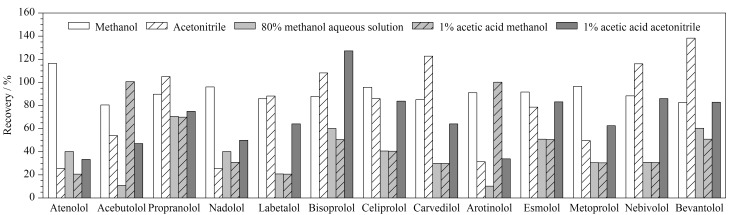
不同提取溶剂对13种*β*-受体阻滞剂回收率的影响

### 2.3 基质效应考察

选择典型空白样品基质,制备质量浓度为20 μg/L的空白基质加标溶液,上机测定,制备同样浓度的标准品溶液,通过二者响应强度的百分比来确定基质效应^[24]^。结果表明,13种*β*-受体阻滞剂的基质效应在87.4%~107.3%范围内,空白基质对目标物定量影响较小。

### 2.4 灵敏度、准确度和精密度

以质量浓度(μg/L)为横坐标,峰面积为纵坐标制作标准曲线,13种*β*-受体阻滞剂在0.5~100 μg/L范围内呈良好的线性关系,相关系数(*r*)均≥0.9912。向空白样品中添加逐级稀释的标准品溶液,经前处理后,上机测定,确定有信号检出的最低含量为该目标物的检出限,为1~10 μg/kg,详细结果见表2。

**表 2:** 13种*β*-受体阻滞剂的回归方程、相关系数、检出限和定量限

Compound	Regression equation	r	LOD/ (μg/kg)	LOQ/ (μg/kg)
Atenolol	y=2.31×10^5^x-4.81×10^5^	0.9970	4	10
Nadolol	y=4.91×10^5^x+5.50×10^5^	0.9912	10	20
Acebutolol	y=6.08×10^5^x+1.10×10^6^	0.9937	2	5
Arotinolol	y=4.89×10^5^x-7.25×10^5^	0.9957	1	3
Metoprolol	y=6.97×10^5^x-6.26×10^5^	0.9930	1	3
Celiprolol	y=7.24×10^5^x-7.98×10^5^	0.9962	1	3
Esmolol	y=9.86×10^5^x-6.33×10^5^	0.9958	4	10
Labetalol	y=4.58×10^5^x-8.50×10^5^	0.9942	1	3
Bisoprolol	y=6.96×10^5^x-6.59×10^5^	0.9957	1	3
Propranolol	y=1.01×10^6^x-6.70×10^5^	0.9951	1	3
Bevantolol	y=7.50×10^5^x-7.39×10^5^	0.9960	1	3
Carvedilol	y=6.72×10^5^x-5.65×10^5^	0.9972	1	3
Nebivolol	y=5.24×10^5^x-7.82×10^5^	0.9956	1	3

*y*: peak area; *x*: mass concentration, μg/L.

分别称取空白样品各1 g,在低、中、高3个水平下分别添加13种*β*-受体阻滞剂标准物质进行加标回收试验,每个水平进行6次平行分析,回收率结果见表3。13种*β*-受体阻滞剂的平均回收率为75.3%~108.4%,相对标准偏差(RSD)为0.9%~10.0%(*n*=6),其准确度和精密度能满足保健食品检测的要求,表明该分析方法准确、可靠,适合此类物质的定量分析。

**表 3:** 13种*β*-受体阻滞剂的加标回收率及相对标准偏差(*n*=6)

Compound	Added/(μg/kg)	Recovery/%	RSD/%
Atenolol	10	105.2	0.9
	20	105.8	4.9
	200	93.8	6.5
Nadolol	20	78.4	3.6
	50	77.8	4.1
	200	78.0	3.1
Acebutolol	10	79.4	4.4
	20	82.8	5.7
	200	81.0	7.1
Arotinolol	10	80.6	3.6
	20	77.2	2.9
	200	76.0	5.1
Metoprolol	10	91.9	4.2
	20	89.6	2.5
	200	102.2	5.6
Celiprolol	10	86.5	4.4
	20	75.3	5.7
	200	81.7	4.4
Esmolol	10	80.9	10.0
	20	95.9	8.6
	200	88.9	2.7
Labetalol	10	87.5	1.5
	20	104.8	5.2
	200	79.6	6.1
Bisoprolol	10	91.8	6.5
	20	108.4	5.9
	200	89.0	2.8
Propranolol	10	86.6	8.2
	20	78.3	1.8
	200	82.0	5.1
Bevantolol	10	89.3	3.4
	20	84.0	3.6
	200	84.8	6.3
Carvedilol	10	78.9	9.4
	20	77.3	4.1
	200	77.6	4.8
Nebivolol	10	89.0	2.5
	20	81.3	1.9
	200	90.3	1.2

### 2.5 *β*-受体阻滞剂类化合物的质谱裂解规律

*β*-受体阻滞剂是一类以苯乙醇胺结构为母核、苯环连接碱性*β*-轻胺(仲胺)的苯乙胺类药物。本实验选用的*β*-受体阻滞剂为芳氧丙醇胺类化合物,含有以N为中心的碱性基团。

由表1中各化合物的二级碎片离子可见,阿替洛尔、醋丁洛尔、普萘洛尔、比索洛尔、艾司洛尔和美托洛尔均产生了*m/z* 116.1070和*m/z* 98.0965的碎片离子,推断其裂解规律为这6种化合物在碳氧键处断裂失去其芳香结构,形成碎片离子C_6_H_14_NO^+^(*m/z* 116.1070),再进一步脱水得到碎片离子C_6_H_12_N^+^(*m/z* 98.0965),以醋丁洛尔为例,其二级质谱裂解机理如图3所示。卡维地洛和贝凡洛尔因含有电负极性较强的间苯二醚结构极易从中间断裂,以卡维地洛为例,其二级质谱裂解机理如图4所示。塞利洛尔、纳多洛尔、拉贝洛尔、阿罗洛尔及萘必洛尔分子结构式中的特征官能团与其他目标物虽然各不相同,但其裂解规律相似。

**图3:**
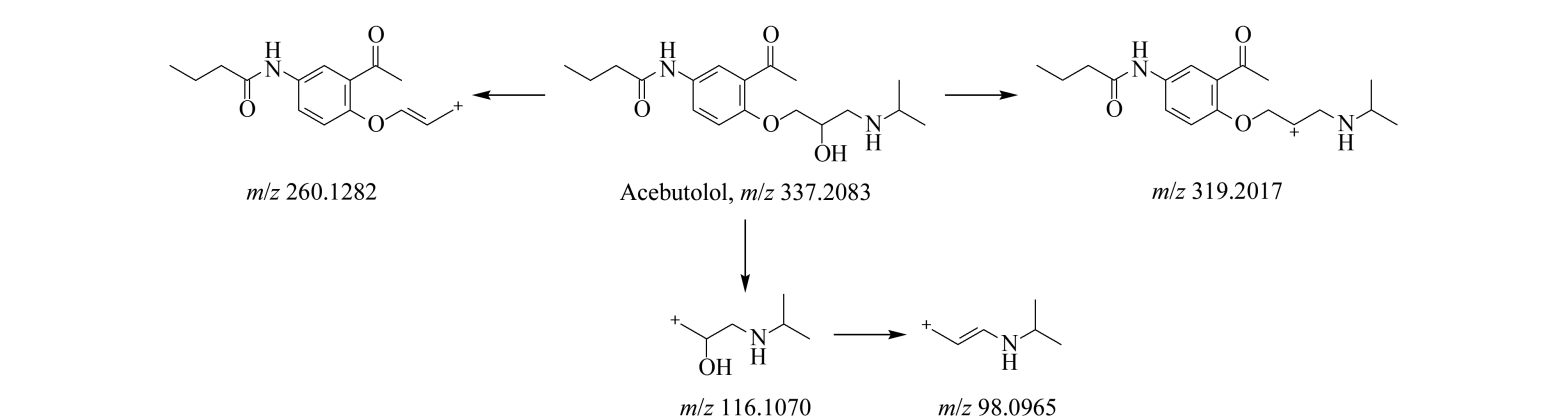
醋丁洛尔二级质谱裂解机理

**图4:**
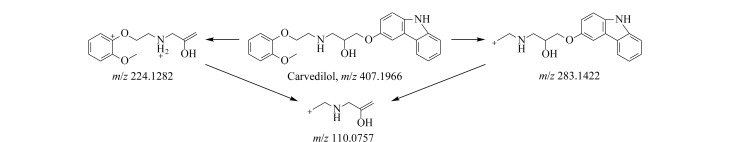
卡维地洛的二级质谱裂解机理

通过研究*β*-受体阻滞剂的质谱裂解规律,找出其中的特征碎片离子,可以对未知的此类化合物实现非靶标筛查,这也有助于监管部门及时发现该类药物的结构类似物在保健食品中的非法添加。

### 2.6 实际样品测定

利用本方法对随机抽取的30批次市售片剂、口服液类保健食品进行快速筛查检测,均未检出上述13种*β*-受体阻滞剂。

## 3 结论

采用超高效液相色谱-四极杆/静电场轨道阱质谱技术,建立了食品中13种*β*-受体阻滞剂的快速精准检测方法,与目前所报道的方法相比,本方法不仅灵敏度满足要求,同时抗干扰能力强,速度快,成本低,可应用于实际样品中非法添加*β*-受体阻滞剂类药物的快速筛查,为监管部门提供了有效的技术支撑。

## References

[1] Chen D Y, Zhang H, Feng J L, et al. Chinese Journal of Chromatography, 2020, 38(8): 880 34213179 10.3724/SP.J.1123.2019.12017

[2] Zhou Y L, Xi Z, Kang J, et al. Journal of Food Safety and Quality, 2022, 13(9): 2908

[3] Sung I K, Park S J, Karig K, et al. Korean J Food Sci Anim Resour, 2015, 135(1): 121 10.5851/kosfa.2015.35.1.121 PMC4682509 26761809

[4] Regal P, Monica D B, Rocio B, et al. Food Addit Contam, 2017, 34(4): 10

[5] Cao Y C, Feng Y X. Journal of Huaihai Medicine, 2019, 39(6): 659

[6] Fan W S, Zhang L, Zhu J K, et al. Occupation and Health, 2023, 39(7): 904

[7] Yue H X, Lei W, Du X N, et al. Journal of Chinese Mass Spectrometry Society, 2018, 39(1): 61

[8] Liu X J, Zhang Y Y, Liu T T, et al. Chemical Analysis and Meterage, 2023, 32(4): 63

[9] Peng J W, Xu L, Li X C, et al. Chemical Industry Times, 2016, 30(8): 1

[10] Wang J J, Zheng G J, Wang J L, et al. Chinese Journal of Analytical Chemistry, 2001, 29(6): 671

[11] Tan X P. [MS Dissertation]. Chongqing: Southwest University, 2016

[12] Cai W J, Ying Y F, Lu C B, et al. Chinese Journal of Veterinary Drug, 2017, 51(8): 26

[13] Hu S J, Li Y, Zhou Y, et al. Chinese Journal of Chromatography, 2019, 37(7): 701 31271009 10.3724/SP.J.1123.2019.01046

[14] Zhao K X, Chai M J, Song X, et al. Journal of Food Safety and Quality, 2019, 10(11): 3481

[15] Liu X H, Shi Y H, Ding W H, et al. Quality and Safety of Agro-products, 2020(5): 68

[16] Yuan L J, Zong W. Journal of Food Safety and Quality, 2022, 13(20): 6518

[17] Zeng X, Zhao T T, Liu C S, et al. Journal of Food Science and Technology, 2023, 23(2): 288

[18] Nie X M, Dong X Y, Xu X L, et al. Chinese Journal of Chromatography, 2019, 37(9): 1011 31642307 10.3724/SP.J.1123.2019.01044

[19] Ni Z, Zhang C S, Xie X C. Chinese Journal of Pharmaceutical Analysis, 2019, 39(5): 895

[20] Zhang Q, Zhao P Z, Qu L, et al. Food Science, 2014, 35(20): 202

[21] Wang H, Zhao Y, Liao P, et al. Chemical Journal of Chinese Universities, 2013, 34(3): 556

[22] Liu B, Bao Y, Lang L, et al. Journal of Food Safety and Quality, 2019, 10(6): 1511

[23] Xu H B, Zhang S P, Du R Y, et al. Chinese Journal of Chromatography, 2022, 40(6): 531 35616198 10.3724/SP.J.1123.2021.12009 PMC9404125

[24] Tan H J, Guo C C, Xing S, et al. Chinese Journal of Chromatography, 2019, 37(9): 969 31642301 10.3724/SP.J.1123.2019.02009

